# Exploration of the effects of 66 mitochondria-associated proteins on different cardiomyopathies: A bidirectional 2-sample mendelian randomization study

**DOI:** 10.1097/MD.0000000000042556

**Published:** 2025-05-30

**Authors:** Zehong Peng, Xin Liu, Xi Zhu, Wenzhuo Zhu, Jianglong Wen, Chao Liu, Conghui Li, Lili Zhu

**Affiliations:** a Department of General Practice, The Second Affiliated Hospital of Hunan Normal University/The 921st Hospital of the Chinese People’s Liberation Army Joint Logistics and Security Forces, Changsha, Hunan Province, China; b Department of Special Services, The Second Affiliated Hospital of Hunan Normal University/The 921st Hospital of the Chinese People’s Liberation Army Joint Logistics and Security Forces, Changsha, Hunan Province, China.

**Keywords:** cardiomyopathy, causality, genetic epidemiology, Mendelian randomization, mitochondria-associated protein, mitochondrial DNA copy number.

## Abstract

The aim of this study was to apply bidirectional Mendelian randomization (MR) to assess and investigate the causal associations between mitochondrial DNA copy number (mtDNA-CN), mitochondrial-associated proteins and cardiomyopathy. The mtDNA-CN and MAP from the IEU Open GWAS database were screened for strong associations with 4 different cardiomyopathy-associated single nucleotide polymorphisms (SNPs) in the IEU Open GWAS and Finnish databases, respectively, and causal associations were investigated using the inverse variance weighting method, the MR-Egger regression method, the weighted median method, the weighted mode method, and the simple mode method. method to explore causality; meanwhile, we used the Cochran *Q* test to assess the variability of SNPs. Horizontal pleiotropy of SNPs was examined by MR-Egger regression analysis and MR-PRESSO method. Sensitivity analyses were performed using the “Leave-One-Out (LOO)” method to determine whether the MR results would be interfered by a single SNP. MR analyses of mtDNA-CN, mitochondria-associated proteins, and different cardiomyopathies, respectively, with IVW as the primary analytical method, showed a statistically significant association between mtDNA-CN and pharmacological cardiomyopathy, *P* < .05. Statistical significance was found between 2 mitochondria-associated proteins (dihydrolipoyl dehydrogenase, mitochondrial apoptosis-inducing factor 1) and hypertrophic cardiomyopathy, both with *P* < .05. Six mitochondria-associated proteins (mitochondrial 39S ribosomal protein L33, ribosomal recycling factor, mitochondrial leucine-rich pentatricopeptide repeat motif-containing protein, serine protease high-temperature requirement protease A2, mitochondrial peptide methionine sulfoxide reductase, and mitochondrial input endomembrane transporter enzyme subunit translocase of inner mitochondrial membrane 14) and dilated cardiomyopathy were statistically significant, both with *P* < .05. Screening for 2 mitochondria-associated proteins (nicotinamide adenine dinucleotide dehydrogenase [ubiquinone] flavoprotein 2, and mitochondrial input endomembrane transporter enzyme subunit translocase of inner mitochondrial membrane 14, prot-a-847) and alcoholic cardiomyopathy was statistically significant，*P* < .05. Statistical significance was found between 1 mitochondria-associated protein (mitochondrial peptide chain release factor 1) screened and pharmacological cardiomyopathy, *P* < .05. Sensitivity analyses of all MR results: the Cochran *Q* test, MR-Egger intercept test, MR-Presso global test, and LOO sensitivity test No significant heterogeneity or horizontal pleiotropy was found at any time (all *P* > .05). There were causal associations between mtDNA-CN, mitochondria-associated proteins and cardiomyopathy, and mtDNA-CN and mitochondria-associated proteins had a certain predictive value for the condition and prognosis of patients with cardiomyopathy.

## 1. Introduction

Cardiomyopathies are a diverse group of diseases that can be defined by their etiology (e.g., hereditary or acquired) or cardiac morphology (e.g., dilated or hypertrophic).^[1]^ The most common forms are classified as hypertrophic cardiomyopathy(HCM), dilated cardiomyopathy (DCM), arrhythmogenic cardiomyopathy, left ventricular nitrification insufficiency(LVNC), alcoholic cardiomyopathy (ACM), and drug-induced cardiomyopathy (DICM).^[2–8]^ We know that mitochondria are important organelles involved in cellular energy metabolism, differentiation, proliferation, reprogramming and senescence.^[9,10]^ Since the myocardium relies on high levels of aerobic metabolism to supply blood and energy substrates to various organs in the body, and since the myocardium relies heavily on mitochondrial oxidative phosphorylation (OXPHOS) to satisfy the constant energy requirements for myofilament contraction and electrical function, mitochondria play a plays a key role,^[11]^ Human mitochondrial disease often affects tissues with high energy demands, such as the heart, and it may be associated with cardiomyopathy and early mortality. It has been suggested that some cardiomyopathies are associated with defects in mitochondria-associated protein homeostasis or in the maintenance of mitochondria-associated protein homeostasis,^[12]^ and that disruption of mitochondria-associated protein homeostasis interferes with mitochondrial energy and ATP production, which can directly affect cardiac function. And failure to maintain protein homeostasis may lead to mitochondrial dysfunction and subsequent mitochondrial autophagy or even apoptosis. Nevertheless, the specific mechanistic link between mitochondrial function and the development of cardiomyopathy remains unclear, and a causal relationship has not been clearly established.

With advances in molecular biology and genetics technology, there has been increasing interest in the molecular biology and genetics of the mechanisms underlying mitochondrial dysfunction in cardiomyopathy. Researchers have found that patients with cardiomyopathy are more likely to have mitochondrial genomic instability than healthy individuals, suggesting that mitochondrial genomic instability may play a role in the pathogenesis of cardiomyopathy.^[11]^ Numerous studies have found that mitochondrial dysfunction is associated with the development of cardiomyopathy; however, there is insufficient evidence to establish a causal relationship.^[13,14]^ Prospective, randomized controlled studies are needed to better understand the role of mitochondria in cardiomyopathy. However, obtaining funding for such studies can be very challenging due to ethical and technical constraints. In view of this, MR analysis seems to be a promising approach to study the causal relationship between mitochondrial dysfunction and cardiomyopathy.

MR is a novel genetic epidemiologic approach that utilizes genetic variation and SNPs to infer causal associations between exposures and disease outcomes, providing new insights into the etiology of disease.^[15,16]^ MR provides a robust method for assessing the relationship between risk factors and disease prognosis. The analysis uses SNPs associated with exposure-related genes as instrumental variables (IVs) under specific assumptions to estimate the causal effect of exposure on outcome.^[17]^ MR designs can avoid the potential residual confounding factors and overcome reverse causality bias.^[18]^ Although observational studies can control for known confounders through statistical techniques, the presence of unknown or unmeasured confounders may affect the results.^[19]^ Randomized controlled trials (RCTs) are considered to be the standard epidemiological design development for establishing a direct causal relationship between risk factors on disease and it has the highest level of evidence in evidence-based medicine, however, due to cost, difficulty of implementation, or ethical issues, RCTs are not always feasible.^[20]^ MR compared to traditional epidemiological studies, it can avoid the interference of reverse causality and confounding factors on the accuracy of results, as well as the time-consuming nature of conducting RCTs, the need for expensive scientific research, the poor compliance due to long term follow-up, and the ethical issues of randomized treatment allocation.^[20]^ Therefore, the aim of this study was to investigate the causal relationship between mitochondrial mtDNA-CN, mitochondrial-related proteins and cardiomyopathies using bidirectional 2-sample MR analyses with the aim of establishing the relationship between mitochondrial function and cardiomyopathies through evidence of genetic variants to establish a causal relationship between mitochondrial function and cardiomyopathy, by studying these relationships, to improve our understanding of the pathogenesis and etiology of cardiomyopathy in patients with cardiomyopathy, as well as better understanding and management of some of the risk factors for cardiomyopathy will enable clinicians to prevent and treat the disease.

## 2. Information and methodology

### 2.1. Study design

**Figure 1.**
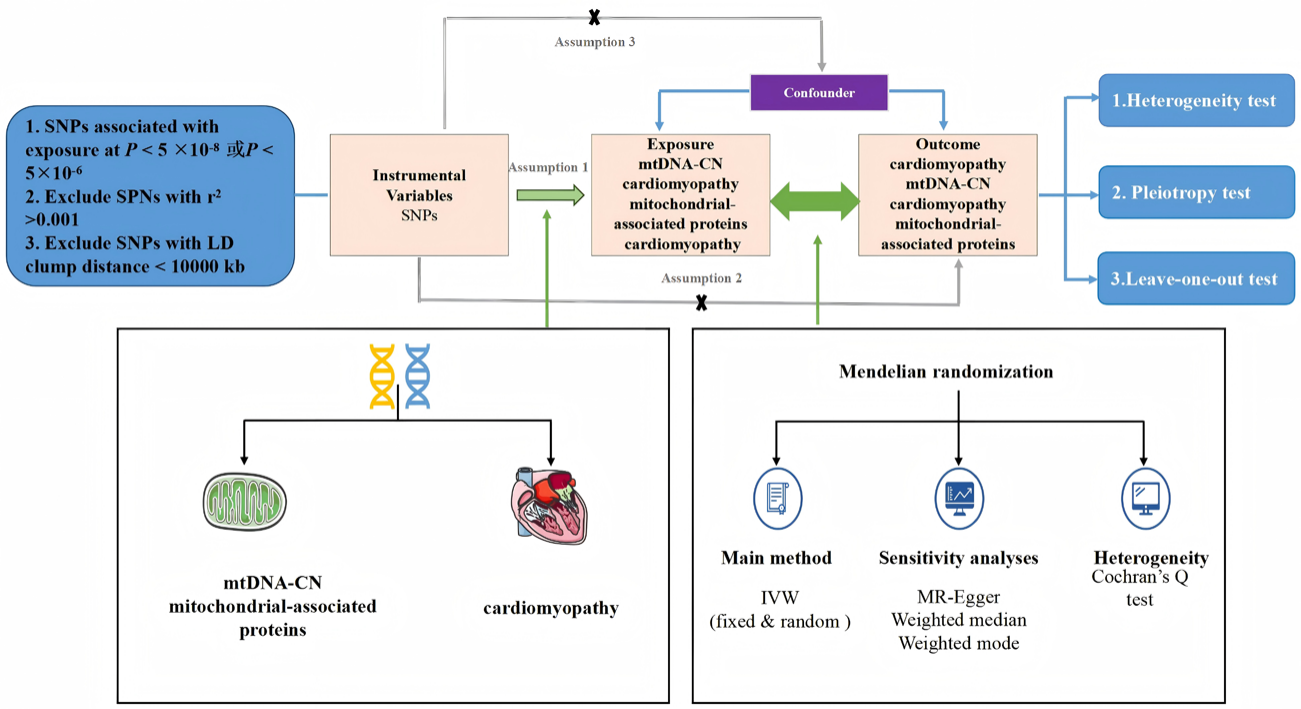
Bidirectional 2-sample MR design for causal analysis of 66 mitochondria-associated proteins and different cardiomyopathies. MR = Mendelian randomization, mtDNA-CN = mitochondrial DNA copy number, SNPs = single nucleotide polymorphisms, IVW = inverse variance-weighted method.

We used a bidirectional 2-sample MR model to assess the causal effects of mtDNA-CN, mitochondria-associated proteins, on cardiomyopathy and used SNPs associated with exposure factors as IVs in the MR study. To perform the MR analyses, the IVs had to satisfy 3 assumptions: relevance assumption: there is a strong association between the exposure factors and the SNPs; independence assumption: there should be no association between the SNPs and the potential confounding factors should not be associated with each other; the exclusivity assumption: the SNPs are only associated with the outcome through exposure. Because this study is a bidirectional 2-sample MR study based on genetic variation, considering that mtDNA encodes mitochondrial proteins, firstly, we performed bidirectional causality between mtDNA-CN and cardiomyopathy, and, secondly, we performed bidirectional causality of mitochondrial-associated proteins on cardiomyopathy as shown in Figure 1.

### 2.2. Data sources

#### 2.2.1. GWAS summary data for mtDNA-CN

The GWAS data of the of mtDNA-CN genetic data were obtained from the IEU Open GWAS database, which is an open access database for genome-wide association studies. mtDNA-CN has a GWAS ID of ebi-a-GCST90026371, a sample size of 383,476 cases, and a number of 24,195,431 SNPs.

#### 2.2.2. GWAS summary data for mitochondria-associated proteins

Genetic data on mitochondria-associated proteins were obtained from The IEU Open GWAS database, a project that provides publicly available summary statistics on 66 mitochondria-associated proteins from 3301 healthy blood donors of European ancestry in the INTERVAL study.^[21]^ All participants provided informed consent in accordance with national research ethics, and demographic characteristics were extensively investigated, including age, sex, anthropometric measures such as height and weight, lifestyle factors (e.g., alcohol consumption and smoking), and dietary habits. In addition, individuals with major medical conditions (e.g., myocardial infarction, stroke, cancer) or recent illnesses or infections were excluded according to blood donation criteria.^[22]^

#### 2.2.3. GWAS summary data for cardiomyopathy

Genetic data on cardiomyopathies were obtained from the IEU Open GWAS database, the Finngen database for 4 different cardiomyopathies (HCM, DCM, ACM, and DICM). Our analysis utilized publicly available GWAS summary data. Each GWAS included in this study received relevant ethical approvals from the respective institutional review boards in the original study, and all participants provided informed consent without the need for ethics committee review.

### 2.3. Instrumental variable screening and data processing

We used different *P*-value thresholds for each extraction of SNPs associated with this exposure. First, for bidirectional causal analysis of mtDNA-CN and cardiomyopathy, *P* < 5 × 10^–8^ was used for forward MR analysis and *P* < 5 × 10^–6^ for reverse analysis; then for bidirectional causal analysis of mitochondria-associated proteins and cardiomyopathy, both forward and reverse MR analyses were used with *P* < 5 × 10^–6^; then to ensure the SNP independence, the chain disequilibrium threshold kb = 10,000, *R*^2^ <0.001, was set, and the parameter was relaxed to *P* < 1 × 10^–5^ when the number of SNPs obtained by screening was less than 2.^[23,24]^ We calculated the value of the *F* statistic by the formula *F*=β^2^/SE^2^, which indicates the strength of association between IVs and exposure factors, using the *F* statistic individual SNPs can be evaluated to confirm their strength in mitigating the potential bias. SNPs with *F* statistic >10 were considered to have a strong strength of association.^[25]^ Prior to MR analyses, data harmonization measures were taken to ensure that the effects of SNPs on exposure and outcome corresponded to the same alleles.^[26]^

### 2.4. MR analysis and sensitivity analysis

In order to select appropriate SNPs as IVs, our study follows the standard assumptions of MR. Five MR analysis methods were used for the 2-sample MR analysis, firstly, the inverse variance-weighted method (IVW) under the random effects model was mainly utilized, which explained the heterogeneity of SNPs.^[27]^ Therefore, after coordinated analysis of GWAS between exposure factors and outcome variables or risk factors, we should use IVW as the main MR method to estimate their causal relationships. Meanwhile, MR-Egger regression method (MR-Egger method), weighted median estimator, weighted mode method, and simple mode method were used as complementary methods for MR analysis, with *P* < .05 was considered statistically significant. When the results of IVW method analysis were inconsistent with the results of the supplemental MR analysis methods, the results of IVW analysis were dominant, or when there were insufficient SNPs that were strongly correlated at the same time, the results of IVW analysis were dominant. Although the inclusion of multiple variants in MR analyses increases statistical efficacy, it introduces the possibility of pleiotropy, where a single genetic variant is associated with multiple variables. We therefore used the MR-Egger regression analysis method and the MR-PRESSO method to detect and correct for any potential horizontal pleiotropy outliers.^[28]^ We used these sensitivity analysis methods to comprehensively assess the robustness of the MR analysis results, thereby mitigating potential bias effects and enhancing the credibility of causal inferences. To assess SNP heterogeneity, we performed Cochran *Q* test and relied on the results of the IVW model in the presence of heterogeneity.^[29]^ In addition, a leave-one-out (LOO) sensitivity test was performed to investigate the likelihood of causality being driven by a single genetic variant.^[30]^ A significance level of *P* < .05 was considered to be indicative of significant het.^[31]^

### 2.5. Statistical analysis

All statistical analyses were performed using the “TwoSampleMR” package and the “Rpackage package (version 0.5.11)” in R Studio (version 4.4.1). The “ggplot2” package was used for data visualization.^[32,33]^ The results of MR were expressed by Odds Ratio (OR), 95% Confidence Intervals (95% CI) and *P*-values. The scatterplot, circumferential heat map, forest plot, funnel plot, and LOO plot allow us to visualize the output of MR intuitively. *P* < .05 is considered statistically significant.

## 3. Results

### 3.1. Bidirectional causality between mtDNA-CN and cardiomyopathy

#### 3.1.1. Forward causal relationship between mtDNA-CN and cardiomyopathy

In the Forward MR analysis with mtDNA-CN as an exposure factor, the results of MR analysis by mitochondrial DNA copy number and 4 different cardiomyopathies with IVW as the main analysis method showed that mtDNA-CN was screened for a forward causative association with pharmacological cardiomyopathy according to the predefined screening criteria (OR = 34.673, 95% CI: 3.192–376.651, *P* = .004), and identified 56 SNPs that were closely related to mtDNA-CN, as shown in Table 1, Table S1 (sheet 9), Supplemental Digital Content, https://links.lww.com/MD/O973, Figure F1 of Figure S1, Supplemental Digital Content, https://links.lww.com/MD/O974; as the OR value was >1, this indicated that mitochondrial DNA copy number might be a risk factor for drug-induced cardiomyopathy and might increase the risk of drug-induced cardiomyopathy; after MR-Egger regression, weighted median, simple model, and weighted model analyses were performed, the results were consistent with the IVW analysis results in the direction of the total effect value, see Table S1 (sheet 9), Supplemental Digital Content, https://links.lww.com/MD/O973, Figure F1 of Figure S1, Supplemental Digital Content, https://links.lww.com/MD/O974. Meanwhile, the results of the forward MR analyses found that there was no significant causal relationship (all *P* > .05), as shown in Table 1 and Table S1 (sheets 6–8), Supplemental Digital Content, https://links.lww.com/MD/O973.

**Table 1:** Major forward MR findings between mtDNA-CN and cardiomyopathy.

Exposure	Exposure ID numbers	Outcome	Number of SNP	IVW		
				OR (95% CI)	*P*	Beta (SE)
mtDNA-CN	ebi-a-gcst90026371	Drug-induced cardiomyopathy	56	34.67 (3.192–376.651)	.004	3.546 (1.217)
mtDNA-CN	ebi-a-gcst90026371	Hypertrophic cardiomyopathy	58	0.825 (0.400–1.700)	.602	−0.192 (0.369)
mtDNA-CN	ebi-a-gcst90026371	Dilated cardiomyopathy	58	0.894 (0.437–1.351)	.631	−0.112 (0.233)
mtDNA-CN	ebi-a-gcst90026371	Alcoholic cardiomyopathy	56	0.833 (0.173–4.011)	.820	−0.182 (0.802)

95% CI = 95% confidence intervals, IVW = inverse variance-weighted method, mtDNA-CN = mitochondrial DNA copy number, OR = odds ratio, SE = standard error.

#### 3.1.2. Forward MR sensitivity analysis between mtDNA-CN and cardiomyopathy

In the sensitivity analysis, no significant heterogeneity or horizontal pleiotropy was found at the Cochran *Q* test, the MR-Egger intercept test, the MR-Presso global test, and the LOO sensitivity test (all *P *> .05), as shown in Table 2, see Table S1 (sheet 11), Supplemental Digital Content, https://links.lww.com/MD/O973, and Figure S1, Supplemental Digital Content, https://links.lww.com/MD/O974.

**Table 2:** Sensitivity analysis of major forward MR findings between mtDNA-CN and cardiomyopathy.

Exposure	Exposure ID numbers	Outcome	Heterogeneity test			Horizontal multiple validity test	
			*Q*-statistic	*P*	Egger-intercep *P*	MR-PRESSO	Global test *P*
mtDNA-CN	ebi-a-gcst90026371	Drug-induced cardiomyopathy	57.788	.373	–.050	0.454	.353
mtDNA-CN	ebi-a-gcst90026371	Hypertrophic cardiomyopathy	59.363	.389	–.011	0.604	NA
mtDNA-CN	ebi-a-gcst90026371	Dilated cardiomyopathy	60.454	.352	–.013	0.306	NA
mtDNA-CN	ebi-a-gcst90026371	Alcoholic cardiomyopathy	46.292	.792	–.082	0.072	NA

MR = Mendelian randomization, mtDNA-CN = mitochondrial DNA copy number.

#### 3.1.3. Reverse causality between mtDNA-CN and cardiomyopathy

In the reverse MR analysis with mtDNA-CN as an exposure factor, the results of MR analysis by mitochondrial DNA copy number and 4 different cardiomyopathies with IVW as the primary analysis method showed that, according to the predefined screening criteria, no significant causative relationship was found between HCM, DCM, ACM, DICM, and mtDNA-CN(all *P *> .05), as shown in Table S2 (sheets 6–9), Supplemental Digital Content, https://links.lww.com/MD/O975.

#### 3.1.4. Inverse MR sensitivity analysis between mtDNA-CN and cardiomyopathy

In the sensitivity analysis, no significant heterogeneity or horizontal pleiotropy was found in the Cochran *Q* test, MR-Egger intercept test, MR-Presso global test and LOO sensitivity test (all *P *> .05), see Table S2 (sheets 10–13), Supplemental Digital Content, https://links.lww.com/MD/O975.

### 3.2. Bidirectional causality between mitochondria-associated proteins and cardiomyopathy

#### 3.2.1. Forward causal relationship between mitochondria-related proteins and cardiomyopathy

In the forward MR analysis with mitochondria-associated proteins as an exposure factor, the results of the MR analysis of 66 mitochondria-associated proteins and different cardiomyopathies with IVW as the main analytical method showed that a total of mitochondria-associated proteins were screened for aForwardcausality with 4 cardiomyopathies based on the preset screening criteria as shown in Table 3, and in Table S3, Supplemental Digital Content, https://links.lww.com/MD/O976.

**Table 3:** Major forward MR findings between mitochondria-associated proteins and cardiomyopathy.

Exposure		Outcome	Number of SNP	IVW		
Mitochondria-associated protein ID	Annotation			OR (95% CI)	*P*	Beta (SE)
prot-a-825	Dihydrolipoyl dehydrogenase	Hypertrophic cardiomyopathy	22	0.821 (0.684–0.985)	.034	–0.198 (0.093)
prot-a-64	Apoptosis-inducing factor 1	Hypertrophic cardiomyopathy	6	1.447 (1.027–2.039)	.034	0.370 (0.175)
prot-a-1942	39S ribosomal protein L33	Dilated cardiomyopathy	11	0.906 (0.831–0.988)	.025	–0.099 (0.044)
prot-a-1945	Ribosome-recycling factor	Dilated cardiomyopathy	14	1.182 (1.002–1.396)	.048	0.168 (0.085)
prot-a-1783	Leucine-rich PPR motif-containing protein	Dilated cardiomyopathy	15	1.094 (1.005–1.190)	.038	0.090 (0.043)
prot-a-1392	Serine protease HTRA2	Dilated cardiomyopathy	9	1.261 (1.041–1.527)	.018	0.232 (0.098)
prot-a-1953	Mitochondrial peptide methionine sulfoxide reductase	Dilated cardiomyopathy	11	0.770 (0.609–0.974)	.029	–0.261 (0.120)
prot-a-847	Mitochondrial import inner membrane translocase subunit TIM14	Dilated cardiomyopathy	9	1.204 (1.003–1.445)	.046	0.186 (0.093)
prot-a-2026	NADH dehydrogenase [ubiquinone] flavoprotein 2	Alcoholic cardiomyopathy	6	0.504 (0.255–0.997)	.049	–0.685 (0.348)
prot-a-847	Mitochondrial import inner membrane translocase subunit TIM14	Alcoholic cardiomyopathy	8	2.222 (1.020–4.844)	.045	0.799 (0.398)
prot-a-1965	Peptide chain release factor 1-like	Drug-Induced Cardiomyopathy	10	0.408 (0.186–0.897)	.026	–0.720 (0.462)

95% CI = 95% confidence intervals, HTRA2 = high-temperature requirement protease A2, IVW = inverse variance-weighted method, NADH = nicotinamide adenine dinucleotide, OR = odds ration, PPR = pentatricopeptide repeat, SE = standard error, TIM14 = translocase of inner mitochondrial membrane 14.

**Figure 2.**
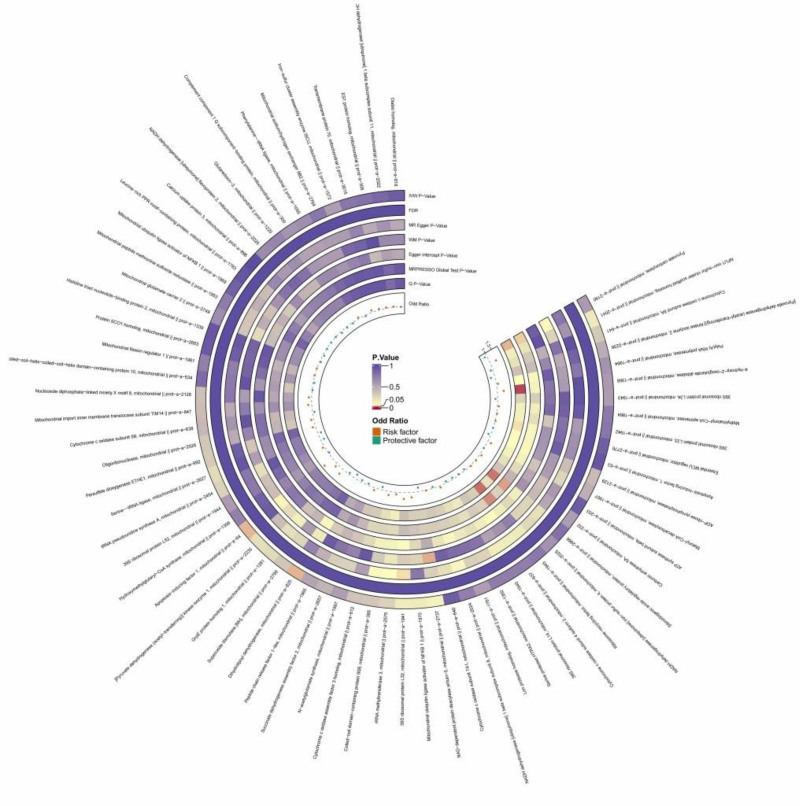
The circular heatmap depicts the results of utilizing MR method to analyze the causal relationship between 66 mitochondrial-associated proteins and hypertrophic cardiomyopathy. MR = Mendelian randomization.

Two mitochondria-associated proteins were found to be statistically significantly associated with HCM, both with *P* < .05, as follows (dihydrolipoyl dehydrogenase, ID No.:prot-a-825, OR = 0.821, 95% CI: 0.684–0.985, *P* = .034); (mitochondrial apoptosis-inducing factor 1, ID number: prot-a-64, OR = 1.447, 95% CI: 1.027–2.039, *P* = .034); since the OR is >1, this suggests that mitochondrial apoptosis-inducing factor 1 may be a risk factor, which may increase the risk of hypertrophic cardiomyopathy; and since the OR is <1, this suggests that dihydrolipoic acid acyl dehydrogenase may be a protective factor and may decrease the risk of hypertrophic cardiomyopathy, see Figure 2, Figure F1 of Figure S2, Supplemental Digital Content, https://links.lww.com/MD/O974, and Table S3 (sheet 2), Supplemental Digital Content, https://links.lww.com/MD/O976.

**Figure 3.**
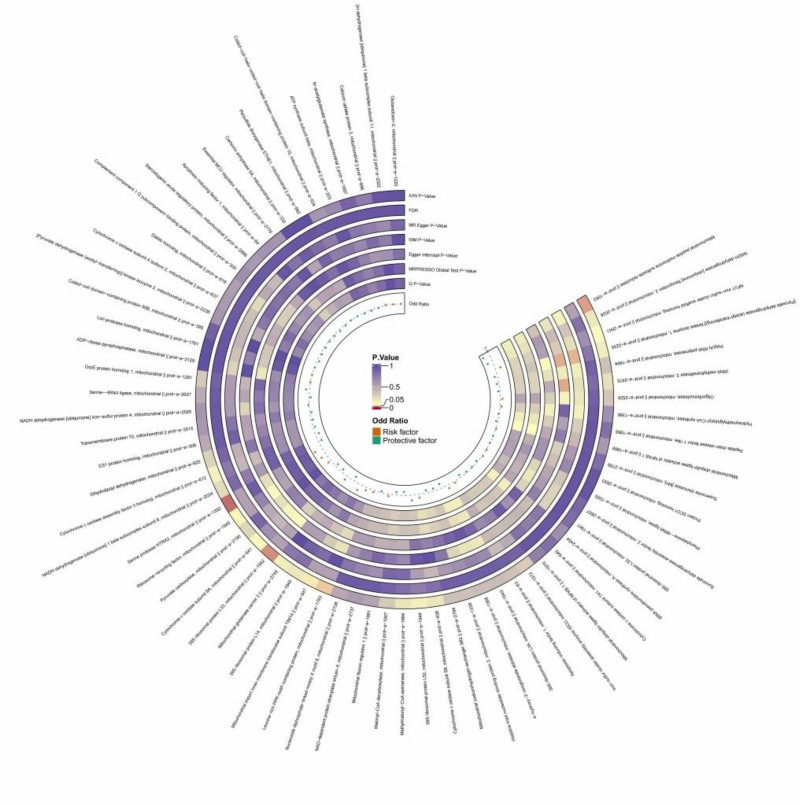
The circular heatmap depicts the results of utilizing MR method to analyze the causal relationship between 66 mitochondrial-associated proteins and dilated cardiomyopathy. MR = Mendelian randomization.

Six mitochondria-associated proteins were found to be statistically significantly associated with DCM with *P* < .05, specifically (mitochondrial 39S ribosomal protein L33, ID No. prot-a-1942, OR = 0.906, 95% CI: 0.831–0.988, *P* = .025), (ribosomal recycling factor, ID No. prot-a-1945, OR = 1.182, 95% CI: 1.002–1.396, *P* = .048), (mitochondrial leucine-containing-rich pentatricopeptide repeat (PPR) motif protein, ID number: prot-a-1783, OR = 1.094, 95% CI: 1.005–1.190, *P* = .038), (serine protease high-temperature requirement protease A2 (HTRA2), ID number: prot-a-1392, OR = 1.261, 95% CI: 1.041–1.527, *P* = .018), (mitochondrial peptide methionine sulfoxide reductase, ID number: prot-a-1953, OR = 0.770, the 95% CI: 0.609–0.974, *P* = .029), (mitochondrial input inner membrane transporter enzyme subunit translocase of inner mitochondrial membrane 14 (TIM14), ID No.: prot-a-847, OR = 1.204, 95% CI: 1.003–1.445, *P* = .046); as the OR is >1, this suggests that ribosomal recycling factors, mitochondrial-containing leucine-rich PPR motif protein, serine protease HTRA2, and mitochondrial input endosomal transporter enzyme subunit TIM14 may be risk factors, which may increase the risk of dilated cardiomyopathy; since the OR is <1, this suggests that mitochondrial 39S ribosomal protein L33, and mitochondrial peptide methionine sulfoxide reductase may be protective factors, which may decrease the risk of dilated cardiomyopathy, as shown in Figure 3, Figure F2 of Figure S2, Supplemental Digital Content, https://links.lww.com/MD/O977 and Table S3 (sheet 3), Supplemental Digital Content, https://links.lww.com/MD/O976.

**Figure 4.**
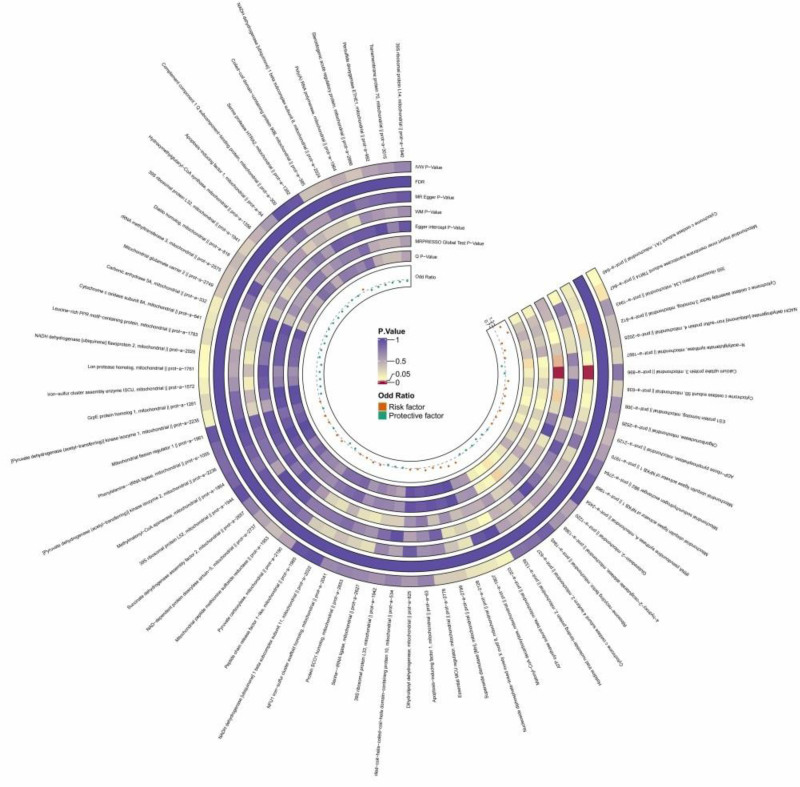
The circular heatmap depicts the results of utilizing MR method to analyze the causal relationship between 66 mitochondrial-associated proteins and alcoholic cardiomyopathy. MR = Mendelian randomization.

Two mitochondria-associated proteins were found to be statistically significantly associated with ACM, both with *P* < .05, specifically (nicotinamide adenine dinucleotide dehydrogenase [ubiquinone] flavoprotein 2, ID No.: prot-a-2026, OR = 0.504, 95% CI: 0.255–0.997, *P* = .049), (mitochondrial input endosomal transporter enzyme subunit TIM14, ID number: prot-a-847, OR = 2.222, 95% CI: 1.020–4.844, *P* = .045); since the OR is >1, this suggests that the mitochondrial input endosomal transporter enzyme subunit TIM14 may be a risk factor that may increase the risk of alcoholic cardiomyopathy; since the OR is <1, this suggests that nicotinamide adenine dinucleotide dehydrogenase [ubiquinone] flavoprotein 2 may be a protective factor and may decrease the risk of alcoholic cardiomyopathy, see Figure 4, Figure F3 of Figure S2, Supplemental Digital Content, https://links.lww.com/MD/O977, and Table S3 (sheet 4), Supplemental Digital Content, https://links.lww.com/MD/O976.

**Figure 5.**
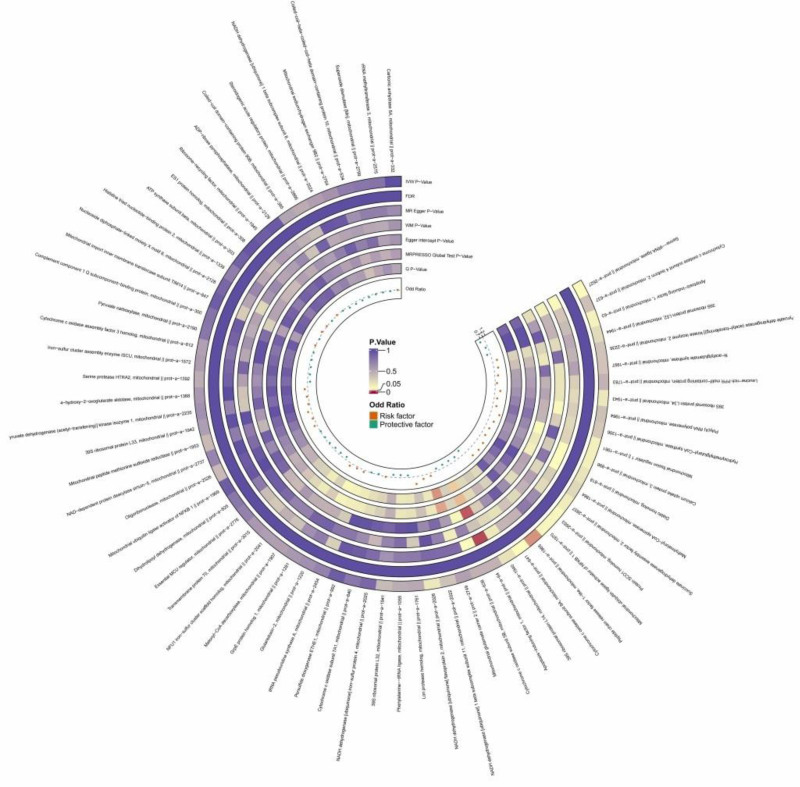
The circular heatmap depicts the results of utilizing MR method to analyze the causal relationship between 66 mitochondrial-associated proteins and drug-induced cardiomyopathy.

One mitochondria-associated protein was found to be statistically significantly associated with DICM with *P* < .05, specifically (mitochondrial peptide chain releasing factor 1, ID No. prot-a-1965, OR = 0.408, 95% CI: 0.186–0.897, *P* = .026); as the OR was <1, this suggests that mitochondrial peptide chain releasing factor 1 may be a protective factor that may reduce the risk of drug-induced cardiomyopathy; since the OR is <1, this suggests that mitochondrial peptide chain releasing factor 1 may be a protective factor and may reduce the risk of drug-induced cardiomyopathy, see Figure 5, Figure F4 of Figure S2, Supplemental Digital Content, https://links.lww.com/MD/O977, Table S3 (sheet 5), Supplemental Digital Content, https://links.lww.com/MD/O976.

#### 3.2.2. Forward MR sensitivity analysis between mitochondria-related proteins and cardiomyopathy

In the sensitivity analysis, no significant heterogeneity or horizontal pleiotropy was found in the Cochran *Q* test, MR-Egger intercept test, MR-Presso global test and LOO sensitivity test (all *P* > .05), see Table 4 and Tables S3 (sheets 6–13), Supplemental Digital Content, https://links.lww.com/MD/O976, and Figure S2, Supplemental Digital Content, https://links.lww.com/MD/O977.

**Table 4:** Sensitivity analysis of major forward MR findings between mitochondria-associated proteins and cardiomyopathy.

Exposure		Outcome	Heterogeneity test				Horizontal multiple validity test
Mitochondria-associated protein ID	Annotation		*Q*-statistic	*P*	Egger-intercep *P*	MR-PRESSO	Global test *P*
prot-a-825	Dihydrolipoyl dehydrogenase	Hypertrophic cardiomyopathy	13.686	.883	–.067	0.080	.897
prot-a-64	Apoptosis-inducing factor 1	Hypertrophic cardiomyopathy	3.004	.699	.073	0.771	.688
prot-a-1942	39S ribosomal protein L33	Dilated cardiomyopathy	5.553	.851	–.028	0.281	.796
prot-a-1945	Ribosome-recycling factor	Dilated cardiomyopathy	7.138	.895	.021	0.522	.928
prot-a-1783	Leucine-rich PPR motif-containing protein	Dilated cardiomyopathy	11.988	.607	.046	0.230	.528
prot-a-1392	Serine protease HTRA2	Dilated cardiomyopathy	3.057	.931	.057	0.558	.943
prot-a-1953	Mitochondrial peptide methionine sulfoxide reductase	Dilated cardiomyopathy	15.091	.129	.074	0.362	.141
prot-a-847	Mitochondrial import inner membrane translocase subunit TIM14	Dilated cardiomyopathy	5.034	.754	.054	0.344	.769
prot-a-2026	NADH dehydrogenase [ubiquinone] flavoprotein 2	Alcoholic cardiomyopathy	2.061	.841	–.046	0.754	.886
prot-a-847	Mitochondrial import inner membrane translocase subunit TIM14	Alcoholic cardiomyopathy	7.781	.352	–.210	0.429	.481
prot-a-1965	Peptide chain release factor 1-like	Drug-induced cardiomyopathy	9.204	.419	.218	0.311	.293

HTRA2 = high-temperature requirement protease A2, MR = Mendelian randomization, NADH = nicotinamide adenine dinucleotide, PPR = pentatricopeptide repeat, TIM14 = translocase of inner mitochondrial membrane 14.

#### 3.2.3. Reverse causality between mitochondria-related proteins and cardiomyopathy

When reverse MR analysis of mitochondria-associated proteins and cardiomyopathy was performed, cardiomyopathy was used as the exposure factor and mitochondria-associated proteins were used as the outcome variable; the results of reverse MR analysis showed that hypertrophic cardiomyopathy was found to be associated with 2 mitochondria-associated proteins in a statistically significant manner with *P* < .05 for both of them, specifically (mitochondria 39S ribosomal protein L33, prot-a-1942, *P* = .048), and (phenylalanine-tRNA ligase, prot-a-1055, *P* = .021), as shown in sheet 2 of Table S4, Supplemental Digital Content, https://links.lww.com/MD/O978; alcoholic cardiomyopathy was found to be statistically significantly associated with 1 mitochondrial-associated protein with *P* < .05, specifically (mitochondrial leucine-containing-rich PPR motif protein, prot-a-1783, *P* = .015), see sheet 4 of Table S4, Supplemental Digital Content, https://links.lww.com/MD/O978; no significant causal relationship was found between dilated cardiomyopathy, drug-induced cardiomyopathy, and mitochondrial-associated proteins (all *P* > .05), see Table S4 (sheets 3 and 5), Supplemental Digital Content, https://links.lww.com/MD/O978.

## 4. Discussion

Since mitochondrial DNA copy number encodes mitochondrial-associated proteins, evidence of a causal relationship between mitochondrial DNA copy number and cardiomyopathy may also suggest a potential effect of mitochondrial-associated proteins on cardiomyopathy. Based on this hypothesis, in this study, we successively explored the causal relationship between mtDNA-CN, mitochondrial-associated proteins, and cardiomyopathy by using bidirectional 2-sample MR analysis. We aimed to comprehensively elucidate the potential role of mitochondrial proteins in the pathogenesis of cardiomyopathy. To the best of our knowledge, this is also the first study to comprehensively investigate the relationship between mitochondrial-associated proteins and cardiomyopathy using bidirectional 2-sample MR.

A cardiomyocyte contains thousands of mitochondria, each containing hundreds of mtDNA-CN. The state of mtDNA-CN is more stable in cardiac tissue than in other tissues, which is essential for normal cardiomyocyte function.^[34]^ Thus mitochondria play an important role in cardiomyocyte differentiation and development. mtDNA defects may lead to complex mitochondrial defects, mitochondrial dysfunction, and ultimately impair embryonic heart development.^[35,36]^ In terms of physiological mechanisms, an increase in mtDNA-CN also usually leads to an increase in mitochondrial respiratory chain protein synthesis, which contributes to the efficiency of mitochondrial OXPHOS. In addition, when mtDNA-CN is high enough, mitochondria can efficiently meet the increased energy demand of the organism in a short period of time. Higher mtDNA-CN levels imply that mitochondria have sufficient capacity to participate in reactive oxygen species(ROS) scavenging, thereby reducing the level of oxidative stress in the body, which is mediated by the mitochondrial antioxidant system, which consists mainly of superoxide dismutase and glutathione peroxidase. mtDNA-CN dynamic balance contributes to the maintenance of mitochondrial function, which reduces cellular autophagy and endoplasmic reticulum stress and improves cellular adaptation to cardiomyopathy. However, an unstable state of mtDNA-CN may be involved in the pathological process of cardiomyopathy. Abnormal mtDNA-CN is associated with a variety of factors, such as oxidative stress, cell proliferation, and drug exposure, and leads to aberrant mitochondrial biology, which may in turn induce apoptosis.^[37]^

Some studies have found lower mtDNA-CN and mitochondrial morphological abnormalities suggesting that mitochondrial dysfunction may be associated with the etiology of LVNC.^[34,38]^ However, our MR results showed that higher mtDNA-CN may increase the risk of pharmacological cardiomyopathy, which is somewhat different from previous studies,^[39]^ and of course we cannot exclude the possibility that this is an increase in the proportion of mutant mtDNAs, which results in the emergence of a clinical disease state that becomes more severe.^[11]^ We therefore emphasize the need for individual-based genetic observations, as well as the integration of RCTs of mtDNA-CN associated with cardiomyopathy in future studies to validate and establish a causal relationship between them.^[40]^

It has been suggested that mitochondria-associated protein homeostasis disorders are one of the mechanisms of cardiomyopathy, which disrupts OXPHOS, causes ROS imbalance, and increases mitochondrial autophagy and apoptosis through abnormal protein import, folding, and maturation. Mitochondrial protein genes that play an important role in these processes and are associated with cardiomyopathy include DnaJ heat shock protein family member C19(DNAJC19), mitochondrial import inner membrane translocase subunit TIM16 (MAGMAS), translocase of the inner mitochondrial membrane 50 (TIMM50), mitochondrial intermediate peptidase (MIPEP), X-prolyl-aminopeptidase 3 (XPNPEP3), HtraA serine peptidase 2 (HTRA2), caseinolytic mitochondrial peptidase chaperone subunit B (CLPB) and heat shock 60-kD protein 1 (HSPD1).^[12,41–46]^ In our MR analysis, we also found that there are different possible causal relationships between different mitochondrial proteins and different cardiomyopathies, some positive and some negative, and their possible mechanisms seem to be not quite the same as those associated with different mitochondrial proteins, i.e., specific mitochondrial proteins may play a key role in the pathogenesis of different cardiomyopathies.

HCM is the most common inherited cardiovascular disease.^[47]^ Ranjbarvaziri Mitochondrial dysfunction is considered to be a common pathogenic mechanism in patients with HCM.^[13]^ Our MR results show that mitochondrial proteins associated with hypertrophic cardiomyopathy are both protective and risk factors, and they may play a protective role through the metabolic process of ROS, or they may be associated with a risk factor through altered cardiac substrate utilization and impaired energy supply,^[48–51]^ or indirectly through a possible role of mitochondrial DNA mutations.^[47,52]^ Also, due to the increased metabolic demands of the heart in the state of hypertrophic cardiomyopathy, which leads to further severe disruption of mitochondrial ultrastructure, mitochondria-associated proteins show corresponding structural and functional alterations.^[53,54]^

The etiology of dilated cardiomyopathy includes genetic (primary dilated cardiomyopathy) or acquired factors (secondary dilated cardiomyopathy). In our study, we found the presence of associated mitochondrial proteins that may increase the risk of dilated cardiomyopathy, and the associated mechanism of occurrence may be related to mitochondrial respiratory chain function and the OXPHOS process;^[12,55]^ there is also a concomitant presence of mitochondrial proteins associated with a reduced risk of dilated cardiomyopathy, which may be due to the fact that these mitochondrial proteins increase the efficiency of mitochondrial metabolism and the production capacity of energy, which allows the cells to respond more efficiently to the stresses that develop under hypoxic conditions.^[56,57]^

ACM is the result of prolonged excessive alcohol intake and is usually accompanied by impaired cardiac contractility and function, with defective mitochondrial function, oxidative stress and apoptosis underlying its etiology.^[58,59]^ In our study, we found the presence of relevant mitochondrial proteins that may increase the risk of alcoholic cardiomyopathy, and the related mechanism of occurrence may be related due to the deleterious effects of alcohol on mitochondrial function, which mainly leads to a decrease in the mitochondrial membrane potential, an increase in the level of ROS, and a decrease in the activity of antioxidants;^[60–62]^ and also at the same time, there is the presence of mitochondrial proteins related to a decrease in the risk of alcoholic cardiomyopathy, which may be due to alcohol-induced decreases in enzyme activity in the tricarboxylic acid cycle and the electron transport chain of mitochondria, which in another way suggests that changes in the expression of different types of mitochondrial proteins mediate changes in mitochondrial function, and thus have different causal effects on alcoholic cardiomyopathy.^[63,64]^ Of course due to the deleterious effects of prolonged or high doses of alcohol may also have some effect on mitochondria-associated proteins in cardiomyocytes,^[65,66]^ or may reduce mitochondria-associated protein activity,^[67]^ which also corresponds to our reverse MR causality.

DICM is defined as myocardial damage caused by exposure to certain drugs or medications, where cardiotoxic drugs affecting mitochondria include several widely used anticancer drugs, antiviral compounds, as well as non-pharmacological alcohol, cocaine, methamphetamine, ecstasy, and synthetic cannabinoids. This drug-induced mitochondrial toxicity in cardiomyocytes due to drugs is caused by a variety of mechanisms, including disruption of the mitochondrial respiratory chain, inhibition of important mitochondrial enzymes, loss of mitochondrial membrane potential, and an increase in mitochondrial oxidative/nitrative stress, with mitochondrial oxidative/nitrative modifications being one of the potentially deleterious events that may be triggered, which can ultimately lead to cell death. Our MR results revealed the presence of mitochondrial proteins associated with reduced risk of drug-induced cardiomyopathy, suggesting that mitochondria-associated proteins may be protective against drug-induced cardiomyopathy per se, but due to the toxic effects of some drugs per se, they can lead to oxidative/nitrative modification of mitochondrial proteins, which ultimately contributes to the development of cardiac dysfunction.^[68–70]^

We used bidirectional 2-sample MR for the exploration of possible mechanisms and causality of mtDNA-CN, mitochondria-associated proteins on cardiomyopathy, and this study has several strengths, one of the most important of which is the bidirectional 2-sample MR design. Previously it was mostly impossible to determine whether the observed association between mitochondria-associated proteins and the risk of cardiomyopathy was causal or a residual bias due to incomplete adjustment for established risk factors. Genetic epidemiology, an epidemiology that aims to elucidate the role of genetic determinants in health and disease and their complex interactions with environmental factors, is of great interest for localizing genes with large effect sizes at the individual level.^[71]^ Mendelian randomization is the genetic epidemiology approach.^[72]^ MR largely reduces residual confounders and reverses causality by relying on random assignment of alleles at conception and strengthens the causal inference of the observed association of exposure factors with cardiomyopathy.^[73,74]^ Also compared to traditional epidemiological studies, we used the most recent and complete GWAS data as IVs in this study to maximize the statistical effect and make the findings more convincing. At the same time we conducted a series of complementary and sensitivity analyses, including the use of MR-Egger analysis to determine the reliability of our IVs, such as the use of LOO analyses to enhance the reliability and robustness of the study results.^[75]^

Nonetheless, the study was limited by the fact that we could only access GWAS aggregated statistics and therefore not individual data.^[76]^ We may also have overlooked weak associations, especially in analyses based on IVs that explain a small fraction of the phenotypic variance. We were also unable to correct for sample overlap. This study could not be expanded to achieve a larger sample size to correct for some of the IVs that showed lower efficiency.^[77]^ In addition, this made it difficult to explore potential nonlinearities in some of the associations. In addition, we restricted our analysis to individuals of European ancestry, which reduces the potential bias introduced by demographics but limits the generalizability of our findings to other populations.^[78]^ While our MR analysis suggests an association between mtDNA-CN, mitochondria-associated proteins, and risk of cardiomyopathy, it provides mainly predictive insights without empirical validation, and, while robust for estimating causality, should not be a substitute for RCTs.

In conclusion, through the study of genetic variants, we systematically evaluated the potential causal associations between mtDNA-CN, mitochondria-associated proteins and cardiomyopathy. Our study provides a better understanding of the possible role of mtDNA-CN, mitochondria-associated proteins in the development of cardiomyopathies. With more knowledge in the mtDNA-CN, mitochondria-associated proteins have a predictive value in the condition and prognosis of patients with cardiomyopathies. We provide innovative insights into the genetic epidemiology of the link between mitochondria-associated proteins and cardiomyopathy. The results of the study emphasize the importance of mitochondrial integrity in the pathogenesis of cardiomyopathies. From a clinical perspective, patients with cardiomyopathy are highly susceptible to mitochondrial dysfunction, and these findings emphasize the importance of assessing mitochondrial functional integrity in the management of cardiomyopathy.

## Acknowledgments

We thank all participants in this study. We are grateful to the IEU Open GWAS, FinnGen database teams for providing public access to summary data.

## Author contributions

**Conceptualization:** Zehong Peng, Jianglong Wen, Lili Zhu.

**Data curation:** Zehong Peng, Xi Zhu, Jianglong Wen.

**Formal analysis:** Zehong Peng, Wenzhuo Zhu, Chao Liu, Lili Zhu.

**Funding acquisition:** Zehong Peng, Chao Liu, Lili Zhu.

**Investigation:** Conghui Li.

**Methodology:** Zehong Peng, Xin Liu.

**Project administration:** Lili Zhu.

**Resources:** Zehong Peng, Lili Zhu.

**Software:** Zehong Peng, Wenzhuo Zhu.

**Supervision:** Xi Zhu.

**Validation:** Xi Zhu.

**Visualization:** Zehong Peng, Conghui Li.

**Writing – original draft:** Zehong Peng, Lili Zhu.

**Writing – review & editing:** Xin Liu, Lili Zhu.

## Supplementary Material


