# Supplementary material for: Exploration of the effects of 66 mitochondria-associated proteins on different cardiomyopathies: A bidirectional 2-sample mendelian randomization study
Source: Medicine (Baltimore). 2025 May 30;104(22):e42556. doi: 10.1097/MD.0000000000042556 (PMC12425093; doi:10.1097/MD.0000000000042556)
Supplement: Supplementary file 2 [file medi-104-e42556-s002.docx]

**Supplementary Material**

**Exploration of the effects of 66 mitochondria-associated proteins on different cardiomyopathies: A bidirectional two sample Mendelian randomization study**

**Supplemental Figure** **F1** Forest plots, scatter plots, and sensitivity analyses of the causal relationship between mtDNA-CN and drug-Induced cardiomyopathy


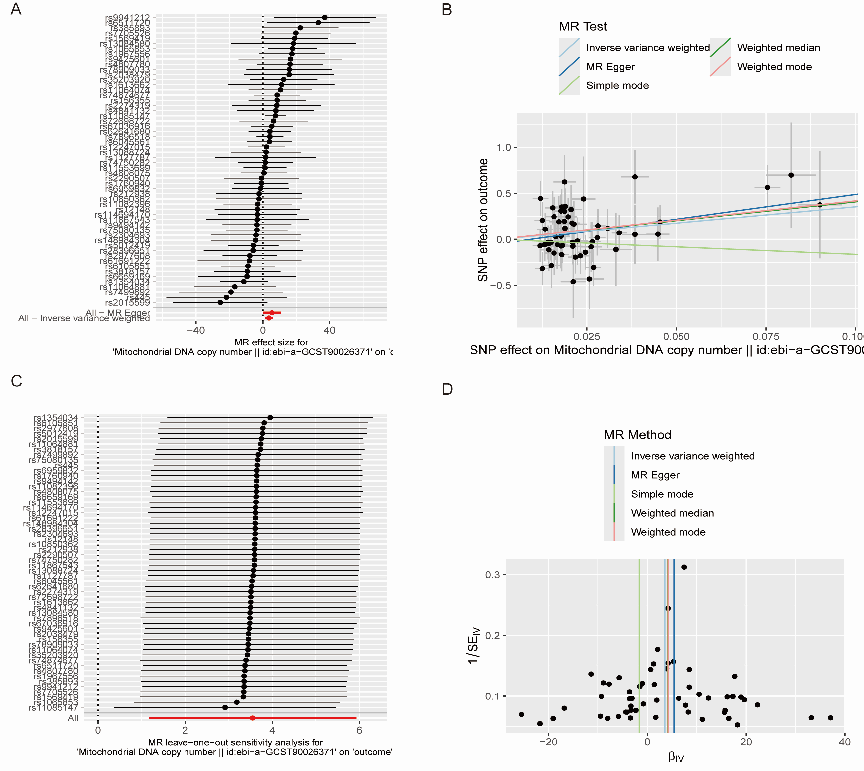


**Figure1** Forest plots, scatter plots, and sensitivity analyses of the causal relationship between mtDNA-CN and drug-Induced cardiomyopathy

A: Forest plot of causal relationship between mtDNA-CN and drug-Induced cardiomyopathy; B: Scatterplot of causal relationship between mtDNA-CN and drug-Induced cardiomyopathy; C: Leave-one-out plot of causal relationship between mtDNA-CN and drug-Induced cardiomyopathy; D: Funnel plot of causal relationship between mtDNA-CN and drug-Induced cardiomyopathy
