# Supplementary material for: Exploration of the effects of 66 mitochondria-associated proteins on different cardiomyopathies: A bidirectional 2-sample mendelian randomization study
Source: Medicine (Baltimore). 2025 May 30;104(22):e42556. doi: 10.1097/MD.0000000000042556 (PMC12425093; doi:10.1097/MD.0000000000042556)
Supplement: Supplementary file 5 [file medi-104-e42556-s005.docx]

**Supplementary Material**

**Exploration of the effects of 66 mitochondria-associated proteins on different cardiomyopathies: A bidirectional two sample Mendelian randomization study**

**Supplemental Figure** **F1 S**catterplot and sensitivity analysis of the causal relationship between mitochondria-associated proteins and hypertrophic cardiomyopathy

**Supplemental Figure F2** Scatterplot and sensitivity analysis plot of the causal relationship between mitochondria-associated proteins and dilated cardiomyopathy

**Supplemental Figure** **F3** Scatterplot and sensitivity analysis plot of the causal relationship between mitochondria-associated proteins and alcoholic cardiomyopathy

**Supplemental Figure F4** Scatterplot and sensitivity analysis plot of the causal relationship between mitochondria-associated proteins and drug-Induced cardiomyopathy


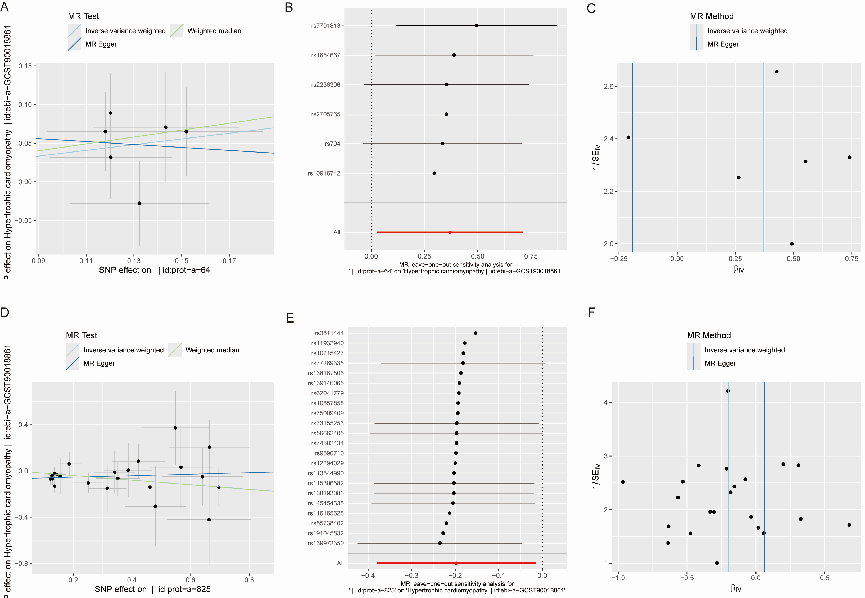


**Figure F1** Scatterplot and sensitivity analysis of the causal relationship between mitochondria-associated proteins and hypertrophic cardiomyopathy

A: scatter plot of causal relationship between mitochondria-associated protein-Dihydrolipoyl dehydrogenase (ID:prot-a-825) and hypertrophic cardiomyopathy; B: leave-one-out plot of causal relationship between mitochondria-associated protein-Dihydrolipoyl dehydrogenase (ID: prot-a-825) and hypertrophic cardiomyopathy; C: leave-one-out plot of causal relationship between mitochondria-associated protein-Dihydrolipoyl dehydrogenase (ID:prot-a-825) and hypertrophic cardiomyopathy Leaving-one-out plot of causality; C: Funnel plot of causality between mitochondria-associated protein-Dihydrolipoyl dehydrogenase (ID:prot-a-825) and hypertrophic cardiomyopathy; D: Funnel plot of causality between mitochondria-associated protein-Apoptosis-inducing factor 1 (ID:prot-a-64) and hypertrophic cardiomyopathy; E: scatter plot of causal relationship between mitochondria-associated protein-Apoptosis-inducing factor 1 (ID No. prot-a-64) and hypertrophic cardiomyopathy; F: leave-one-out plot of causal relationship between mitochondria-associated protein-Apoptosis-inducing factor 1 (ID:prot-a-64) and hypertrophic cardiomyopathy in a funnel plot of causality.
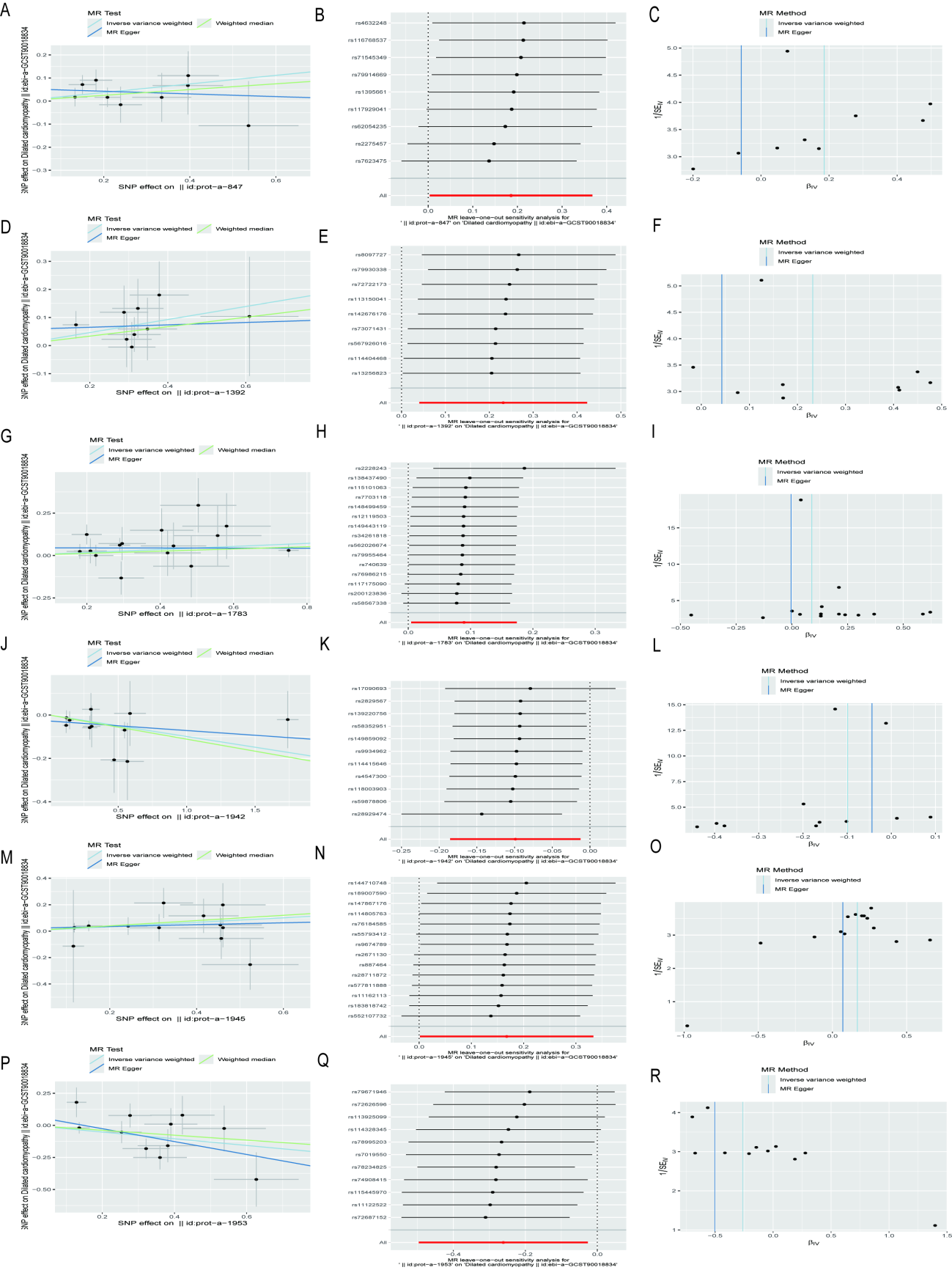
**Figure F2** Scatterplot and sensitivity analysis plot of the causal relationship between mitochondria-associated proteins and dilated cardiomyopathy

A: scatter plot of the causal relationship between mitochondria-associated protein-39S ribosomal protein L33 (ID: prot-a-1942) and dilated cardiomyopathy; B: leave-one-out plot of the causal relationship between mitochondria-associated protein-39S ribosomal protein L33 (ID: prot-a-1942) and dilated cardiomyopathy;C:Funnel plot of the causal relationship between mitochondria-associated protein-39S ribosomal protein L33 (ID:prot-a-1942) and dilated cardiomyopathy;D:Causal relationship between mitochondria-associated protein-Ribosome-recycling factor (ID: prot-a-1945) and dilated cardiomyopathy scatterplot; E: leave-one-out plot of the causal relationship between mitochondria-associated protein-Ribosome-recycling factor (ID: prot-a-1945) and dilated cardiomyopathy; F: funnel plot of the causal relationship between mitochondria-associated protein-Ribosome-recycling factor (ID : prot-a-1945) and dilated cardiomyopathy funnel plot of causality; G: scatter plot of causality between mitochondria-associated protein-Leucine-rich PPR motif-containing protein (ID: prot-a-1783) and dilated cardiomyopathy; H: scatter plot of causality between mitochondria-associated protein-Leucine-rich PPR motif- containing protein (ID: prot-a-1783) and dilated cardiomyopathy causal relationship of leave-one-out plots;I: mitochondria-associated protein-Leucine-rich PPR motif-containing protein (ID: prot-a-1783) and dilated cardiomyopathy causal relationship of funnel Figure; J:Scatterplot of the causal relationship between mitochondria-associated protein-Serine protease HTRA2 (ID:prot-a-1392) and dilated cardiomyopathy; K:Leave-one-out plot of the causal relationship between mitochondria-associated protein-Serine protease HTRA2 (ID:prot-a-1392) and dilated cardiomyopathy; L. Funnel plot of the causal relationship between mitochondria-associated protein-Serine protease HTRA2 (ID: prot-a-1392) and dilated cardiomyopathy; M: scatter plot of causal relationship between mitochondria-associated protein-Mitochondrial peptide methionine sulfoxide reductase (ID: prot-a-1953) and dilated cardiomyopathy; N: leave-one-out plot of causal relationship between mitochondria-associated protein-Mitochondrial peptide methionine sulfoxide reductase (ID:prot-a-1953) and dilated cardiomyopathy; O:funnel plot of mitochondrial-associated protein- Funnel plot of the causal relationship between Mitochondrial peptide methionine sulfoxide reductase (ID: prot-a-1953) and dilated cardiomyopathy; P: Mitochondria-associated protein-Mitochondrial import inner membrane Scatter plot of the causal relationship between translocase subunit TIM14(ID:prot-a-847) and dilated cardiomyopathy;Q: Mitochondrial-associated protein-Mitochondrial import inner membrane translocase subunit TIM14 (ID: prot- a-847) and dilated cardiomyopathy; R:Funnel plot of the causal relationship between mitochondria-associated protein-Mitochondrial import inner membrane translocase subunit TIM14 (ID: prot-a-847) and dilated cardiomyopathy.


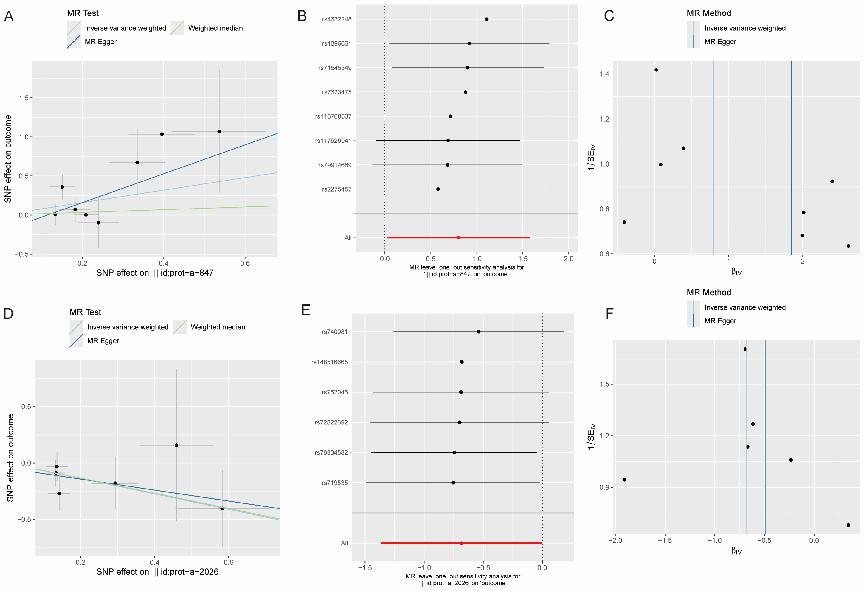


**Figure F3** Scatterplot and sensitivity analysis plot of the causal relationship between mitochondria-associated proteins and alcoholic cardiomyopathy

A: scatter plot of the causal relationship between mitochondria-associated protein-NADH dehydrogenase [ubiquinone] flavoprotein 2 (ID: prot-a-2026) and alcoholic cardiomyopathy; B: leave-one-out plot of the causal relationship between mitochondria-associated protein-NADH dehydrogenase [ubiquinone] flavoprotein 2 (ID:prot-a-2026) and alcoholic cardiomyopathy causal relationship of leave-one-out plot; C: mitochondria-associated protein-NADH dehydrogenase [ubiquinone] flavoprotein 2 (ID:prot-a-2026) and alcoholic cardiomyopathy causal relationship of funnel Figure; D: scatter plot of the causal relationship between mitochondria-associated protein-Mitochondrial import inner membrane translocase subunit TIM14 (ID: prot-a-847) and alcoholic cardiomyopathy; E: mitochondria-associated protein-Mitochondrial import inner membrane translocase subunit TIM14 (ID:prot-a-847) and alcoholic cardiomyopathy; F: mitochondria-associated protein-Mitochondrial import inner membrane translocase subunit Funnel plot of the causal relationship between TIM14 (ID: prot-a-847) and alcoholic cardiomyopathy.


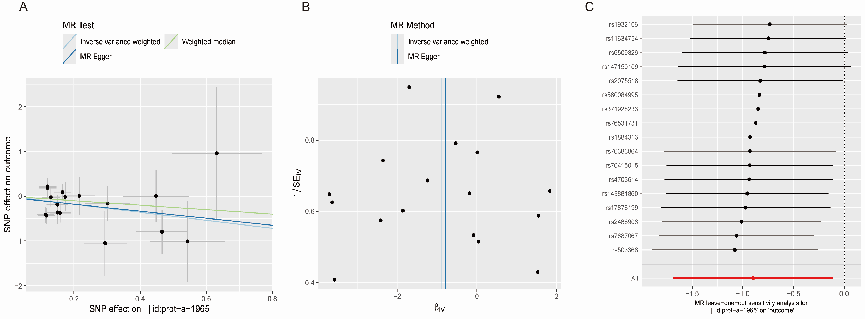


**Figure F4** Scatterplot and sensitivity analysis plot of the causal relationship between mitochondria-associated proteins and drug-Induced cardiomyopathy

A: Scatterplot of the causal relationship between mitochondria-associated protein-Peptide chain release factor 1-like (ID: prot-a-1965) and drug-induced cardiomyopathy; B: leave-one-out plot of the causal relationship between mitochondria-associated protein-Peptide chain release factor 1-like (ID:prot-a-1965) and drug-Induced cardiomyopathy; C: funnel plot of the causal relationship between mitochondria-associated protein-Peptide chain release factor 1-like (ID: prot-a-1965) and drug-Induced cardiomyopathy .
